# “Hard” Drug Repurposing for Precision Oncology: The Missing Link?

**DOI:** 10.3389/fphar.2018.00637

**Published:** 2018-06-14

**Authors:** Pan Pantziarka, Gauthier Bouche, Nicolas André

**Affiliations:** ^1^Anticancer Fund, Brussels, Belgium; ^2^The George Pantziarka TP53 Trust, London, United Kingdom; ^3^Department of Pediatric Hematology-Oncology, Assistance Publique Hôpitaux de Marseille, La Timone Hospital, Marseille, France; ^4^Centre de Recherche en Oncologie Biologique et en Oncopharmacologie, Institut National de la Santé et de la Recherche Médicale-UMR 79 911, Aix-Marseille University, Marseille, France; ^5^Metronomics Global Health Initiative, Marseille, France

**Keywords:** drug repurposing, drug Repositioning, precision oncology, drug development strategy, targeted therapy

Drug repurposing is at the heart of precision oncology. The move from tissue- or cancer-specific treatments to genomic- or actionable-target treatments necessarily entails the reuse of anti-cancer medications licensed for one type of cancer to treat other cancer types (Yates et al., 2018). However, given the genetic heterogeneity of individual cancers, limiting the search of suitable agents to existing oncological drugs limits treatment options. It misses a wide range of possibly useful agents from other disease areas. If we characterize the re-use of existing oncological drugs to new indications as “soft repurposing,” then the use of non-cancer drugs as anti-cancer medications may be termed “hard repurposing” and poses a number of distinct challenges.

The starting point for precision oncology is molecular profiling—based on tumor and germline genomics, DNA sequencing, exome analysis, transcriptomics and other ‘omics (Singer et al., 2017) that is becoming routinely available in many clinical centers. In part this is a function of improvements in technology and concomitant reduction in associated costs. The recent Pan-Cancer Atlas collaboration assessing the combined data from 33 cancer atlas projects reported 299 cancer driver genes and >3400 putative missense driver mutations, it was estimated that only 57% of tumors harbor clinically actionable oncogenic events (Bailey et al., 2018). The move to precision oncology is also increasingly driven by patient request—there is an increased public understanding that genomics offers opportunities to be a gateway to more targeted therapies. Clinicians too are keen to use genomic analysis to avoid over-treatment of patients with toxic drugs which pharmacogenetics or retrospective data analysis suggests is unlikely to benefit them. In the case of EGFR-mutated or ALK-rearranged lung cancer, and BRAF-mutated melanoma, it is now standard practice to use targeted agents where indicated (Barlesi et al., 2016).

For cases where there are no standard treatment options based on molecular subtype, or where resistance has emerged, the challenge is both to identify molecular pathways suspected of “driving” tumor growth and drugs which specifically target those pathways. Such a process requires the input of a wider range of expertise than is common in a traditional multi-disciplinary team meeting—for example bioinformaticians, cancer geneticists, pharmacogeneticists and other non-medical specialists. Typically this process is the remit of a molecular tumor board (MTB), which are being instituted in many cancer centers.

The soft repurposing of existing cytotoxics, endocrine therapies and licensed targeted agents has a number of obvious advantages. These are well-characterized in an oncology setting, with extensive individual and institutional use as cancer treatments, albeit not in the particular cancer types under consideration. However, as shown by the SHIVA trial—a Phase II open-label randomized controlled trial (RCT) of molecularly selected targeted therapies vs. conventional therapy in advanced cancers—the palette of licensed targeted agents is relatively limited compared to the range of driver pathways active in cancer (Le Tourneau et al., 2015). Of 741 screened patients 40% had at least one molecular alteration matching one of the 10 available regimens, 195 (26%) patients were randomly assigned, with 99 in the experimental group and 96 in the control group. The trial failed in its primary outcome as it did not show improvement in progression-free survival (PFS) and also showed that toxicity was comparable in the two arms.

The MOSCATO trial (NCT01566019), successfully biopsied 948 (of 1043 consented) patients with non-curable metastatic cancers and generated genomic analysis for 843 (89%) (Massard et al., 2017). Actionable targets were identified in 411 (43% of biopsied patients), of which 199 were randomized to a molecularly targeted agent. Results showed that the PFS2/PFS1 ratio was >1.3 in 33% of the patients (63/193). Objective responses were observed in 22 of 194 patients (11%; 95% CI, 7–17%), and median overall survival was 11.9 months (95% CI, 9.5–14.3 months). Again selection of agents in this trial was limited to existing targeted drugs.

In contrast the Personalized Oncogenomics Program of British Columbia (NCT02155621), a large (*n* = 5,000) interventional trial includes a wider range of agents, including non-cancer drugs (Laskin et al., 2015). A notable example, is of a patient with metastatic colorectal cancer refractory to standard treatments and suffering from treatment related toxicity (Jones et al., 2016). Whole-genome and transcriptome sequencing followed and integrative molecular analysis indicated genetic and transcriptional overexpression of the *JUN* and *FOS* genes that encode the activating protein-1 (AP-1) complex. Robust c-JUN protein expression was confirmed by immunohistochemistry. These findings led to the hypothesis that blockade of the renin–angiotensin system could be a novel treatment option. A literature screen provided additional data substantiating this hypothesis in the context of colon cancer. The angiotensin II receptor antagonist, irbesartan (known to target AP-1 and listed as such in public databases such as DrugBank), was administered as an anticancer therapy, leading to a dramatic and durable response.

A small cancer-specific trial looked specifically at recurrent glioblastoma (NCT02060890) used a wider panel of agents, including a subset of FDA-approved non-cancer drugs with evidence of blood-brain barrier penetration (Byron et al., 2018). For 16 of 20 enrolled patients the genomic analysis was completed and MTB made treatment recommendations for 15 patients. Five of seven actioned recommendations included repurposed non-cancer drugs and 2 of these patients achieved 12-months PFS of whom one remains progression-free after 21-months.

Much precision oncology work is performed outside of clinical trials—in some centers it is now a standard option for difficult-to-treat patients. The use of case reports is particularly pertinent in such cases, helping to expand our knowledge base to provide supporting evidence to both clinicians and triallists. For example, Cornelius et al report on a case of a 4-month old child with a refractory choroid plexus carcinoma (Cornelius et al., 2017). Molecular profiling identified a combination of sirolimus, thalidomide, sunitinib, and vorinostat as potential therapies. Treatment with the drug cocktail led to 92% reduction in tumor size, no serious adverse events, excellent quality of life and long term survival.

Whether or not generic combinations of repurposed drugs with standard anticancer agents can be used for patients with the same disease is another interesting question. The low toxicity of drug repurposing allows complex combinations that can target several crucial pathways for a given disease and to decrease the risk of resistance observed when using single agent targeted therapy in patients. The recent case report from Berland et al. (2017) confirm previous findings published by Pearl of the potential activity of the MEMMAT combination for atypical teratoid rhabdoid tumors (Peyrl et al., 2012).

The availability of a pool of potentially useful repurposed drugs will be a key determinant of the success of precision oncology. A number of groups have published extensive reviews of possible repurposing targets for specific cancers or groups of cancers, such as AML (Andresen and Gjertsen, 2017). Pantziarka et al. have also curated a list of over 250 non-cancer drugs with anti-cancer potential (Pantziarka et al., 2017). Another challenge is to continuously integrate new findings and to publish results to inform other clinicians and investigators. However, additional bioinformatics work to link molecular pathways and all repurposed agents is clearly warranted.

In addition to providing an expanded pool of active agents, thereby expanding the range of “actionable pathways,” the repurposing of non-cancer drugs, particularly generic medications offer precision oncology options to clinicians in low and middle income countries (Hernandez et al., 2017). Given the high costs of modern drugs, precision oncology based only on these drugs will be unaffordable in most low income settings, and indeed pose strains on health systems even in high income countries. The promise of repurposed drugs is to make precision oncology a reality in all health systems globally (André et al., 2013).

## Author contributions

All authors listed have made a substantial, direct and intellectual contribution to the work, and approved it for publication.

### Conflict of interest statement

The authors declare that the research was conducted in the absence of any commercial or financial relationships that could be construed as a potential conflict of interest.
